# Achieving efficient protein expression in *Trichoderma reesei* by using strong constitutive promoters

**DOI:** 10.1186/1475-2859-11-84

**Published:** 2012-06-18

**Authors:** Junxin Li, Juan Wang, Shaowen Wang, Miao Xing, Shaowen Yu, Gang Liu

**Affiliations:** 1 College of Life Science, Shenzhen Key Laboratory of Microbial Genetic Engineering, Shenzhen University, Shenzhen, 518060, China

**Keywords:** *Trichoderma Reesei*, Xylanase, Pyruvate decarboxylase, Enolase, Quantitative real-time PCR

## Abstract

**Backgrounds:**

The fungus *Trichoderma reesei* is an important workhorse for expression of homologous or heterologous genes, and the inducible *cbh1* promoter is generally used. However, constitutive expression is more preferable in some cases than inducible expression that leads to production of unwanted cellulase components. In this work, constitutive promoters of *T. reesei* were screened and successfully used for high level homologous expression of xylanase II.

**Results:**

The transcriptional profiles of 13 key genes that participate in glucose metabolism in *T. reesei* were analyzed by quantitative real-time reverse-transcription polymerase chain reaction (RT-qPCR). The results indicated that the mRNA levels of *pdc* (encoding pyruvate decarboxylase) and *eno* (encoding enolase) genes were much higher than other genes under high glucose conditions. Recombinant *T. reesei* strains that homologously expressed xylanase II were constructed by using the promoters of the *pdc* and *eno* genes, and they respectively produced 9266 IU/ml and 8866 IU/ml of xylanase activities in the cultivation supernatant in a medium with high glucose concentration. The productivities of xylanase II were 1.61 g/L (with the *pdc* promoter) and 1.52 g/L (with the *eno* promoter), approximately accounted for 83% and 82% of the total protein secreted by *T. reesei*, respectively.

**Conclusions:**

This work demonstrates the screening of constitutive promoters by using RT-qPCR in *T. reesei*, and has obtained the highest expression of recombinant xylanase II to date by using these promoters.

## Backgrounds

*Trichoderma reesei* is an attractive host for the expression of homologous and heterologous proteins because of its ability to secrete large amounts of hydrolytic enzymes [1-3]. It has been reported that highly productive *T. reesei* strains are able to produce and secrete up to 100 g/L of protein in optimal culture conditions, and the main ingredients are cellulases [4]. Of the secreted proteins in *T. reesei*, cellobiohydrolase I (CBHI) dominates, accounting for approximately 50%-60% of the total secreted proteins [5,6]. Since CBHI is encoded by single copy of *cbh1*, the *cbh1* promoter is considered to be strong and has been used to produce various kinds of homologous or heterologous proteins [1,7-10].

The *cbh1* promoter is an inducible promoter. It is induced by several kinds of saccharides, such as cellulose, sophorose, lactose, etc., and regulated by catabolic repression. When the *cbh1* promoter is used for protein expression, an inducer (or inducers) has to be added to trigger the expression of the target genes. However, such inducers also promote the expression of cellulase components, such as cellobiohydrolases, endo-β-glucanases, xylanases, etc. [11-13]. Unselective expression of cellulase components leads to contamination of target proteins with an excess of irrelevant proteins, and increases the difficulty for protein purification. Furthermore, extracellular proteases, which are deleterious to the yield of protein expression, might be produced simultaneously with cellulase induction [4].

In contrast, recombinant protein production mediated by constitutive promoters in *T. reesei* is more selective. Constitutive promoters drive gene expression without inducers. Unlike inducible promoters which are repressed by glucose, most of constitutive promoters are active in a glucose-rich medium. As cellulases whose formation is repressed with high concentration of glucose account for 90%-95% of the *T. reesei* extracellular proteins [14], application of constitutive promoters can effectively reduce the accumulation of irrelevant proteins. Furthermore, in the case of constitutive expression, synthesis of extracellular proteases, which may digest the expressed products, is also inhibited, at least partly, by high glucose concentration [15,16]. Several constitutive promoters of *T. reesei*, such as the *tef1* and *pki* promoters, and the promoter of an unidentified cDNA1, have been employed for recombinant protein production [15,17]. However, the efficiency of these promoters is relatively low. Efforts have been made to convert the *cbh1* promoter into a constitutive promoter by mutating the sequences therein that are responsible for catabolic repression, and the modified *cbh1* promoters that are constitutively active have been obtained. However, the protein expression level is about ten times lower in the presence of glucose than those obtained on a sorbitol-sophorose medium with the wild type *cbh1* promoter [18].

This study describes the screening of strong constitutive promoters and homologous over-expression of xylanase II (XYNII) with these promoters in *T. reesei* QM9414. The *T. reesei* strain is cultivated in a glucose containing medium, and the transcriptional profile of 13 key genes related to glucose metabolism is analyzed by using quantitative real-time reverse-transcription polymerase chain reaction (RT-qPCR). The promoters and the terminators of two genes (*pdc*, encoding pyruvate decarboxylase; and *eno*, encoding enolase) with high expression level are used to construct expression cassettes that consist of XYNII, which are then transformed into the parental strain *T. reesei* QM9414. The recombinant *T. reesei* strains are cultivated in a modified Mandels medium, and extremely high yields of recombinant XYNII are obtained. The highest xylanase activity in the culture supernatant of the recombinant strain is 9266 IU/ml, which is the highest recombinant expression of XYNII achieved to date.

## Results

### Evaluation of constitutive promoter activities in different glucose concentrations

**Figure 1:**
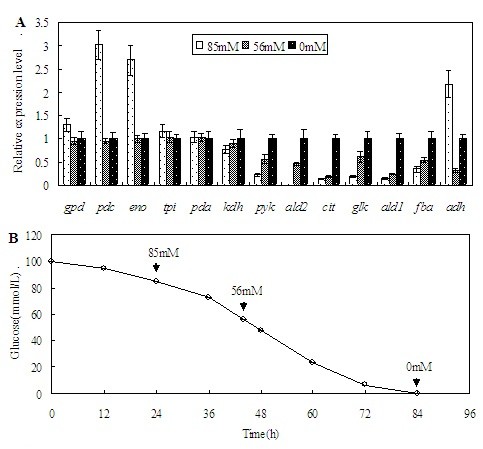
**(A) The time course of glucose consumption in *T. reesei* culture. (B)The transcriptional profiles of the 13 key genes related with glucose metabolism during cultivation.***Error bars* represent standard deviations. The mRNA level of the genes at a 0 mM glucose concentration is assigned 1. The relative expression levels of the genes at 85 mM and 56 mM glucose concentrations are shown in *white dotted bars* and *grey dotted bars*, respectively.

The genes that are responsible for glucose utilization are usually regarded as housekeeping genes, and their expression is driven by constitutive promoters. However, as previously described, the expression of these genes in *T. reesei* might still be affected by glucose concentration [19]. 13 critical genes that participate in glucose metabolism according to Chambergo *et al.*[19] were selected and their transcriptional profiles were quantitatively analyzed by using RT-qPCR. These genes include those encoding glyceraldehyde-3-phosphate dehydrogenase (*gpd*), pyruvate decarboxylase (*pdc*), enolase (*eno*), alcohol dehydrogenase (*adh*), triose phosphate isomerase (*tpi*), aldolase (*fba*), pyruvate kinase *(pyk*), citrate synthase (*cit*), α-ketoglutarate dehydrogenase (*kdh*), aldehyde dehydrogenase I (*ald1*), aldehyde dehydrogenase II (*ald2*), pyruvate dehydrogenase (*pda*), and glucokinase (*glk*), which participate in glycolysis and the tricarboxylic acid cycle. Samples were taken at 24 h, 44 h and 84 h of cultivation, when the residue concentration of glucose was 15.6 g/L (about 85 mM), 10.3 g/L (about 56 mM) and 0 g/L, respectively. The results indicated that the expression levels of two genes (*pdc* and *eno*) decreased in accordance with glucose exhaustion, the expression levels of six genes (*pyk, ald2, cit, glk, ald1* and *fba*) increased with glucose exhaustion, while those of four genes (*gpd, tpi, pda and kdh*) were relatively stable (Figure 1). The expression level of the *adh* gene changed irregularly during glucose exhaustion, possibly due to other factors that influenced the transcription of this gene. To construct a constitutive expression system, the promoters that are active at high glucose concentration might be preferable, for recombinant protein production in rapidly growing cells is more active. The formation of proteases is also somewhat repressed in high-level glucose cultivation [15,16]. Therefore, according to an analysis of the relationship between the transcriptional efficiency of the 13 genes and glucose concentration, the promoters of *pdcenogpd, tpi, pda and kdh* appear to be proper candidates for the construction of a constitutive expression system.

### Selection of strong constitutive promoters

**Figure 2:**
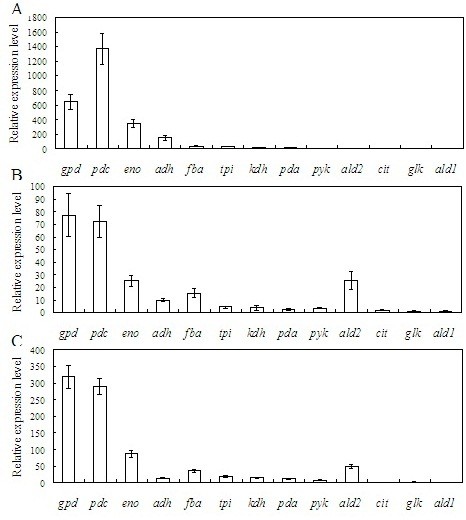
**Comparison of the expression levels of the 13 genes related with glucose metabolism.***Error bars* represent standard deviations. A, B and C indicate the relative expression levels of the genes at 84 h, 44 h, and 24 h of cultivation, when the glucose concentration in the medium was 0 mM, 56 mM and 85 mM, respectively. The mRNA level of *ald1* is assigned 1. *White bars* represent the relative expression levels of the 12 genes.

The relative expression levels of the genes in *T. reesei* QM9414 that grows at different glucose concentrations are shown in Figure 2. The expression levels of *pdcenogpd* are obviously higher than the other genes, especially when *T. reesei* grows at high glucose concentrations. For instance, the mRNA level of *pdc* is approximately 1,373 times higher than that of *ald2* when the glucose concentration is 85 mM. The expression level of the *gpd* gene is the highest among the 13 genes at low glucose concentrations. The mRNA levels of *gpd* are 39 times higher (at a glucose concentration of 56 mM) and 22 times higher (at a glucose concentration of 0 mM) than those of *pyk*, whose promoter has been applied in the construction of a constructive expression system and proven to be relatively weak [17]. Since the expression levels of *pdc* and *eno* increased with glucose concentration, their promoters might be stronger than that of *gpd* at higher glucose concentrations. Actually, at a glucose concentration of 85 mM, the expression level of *pdc* had already surpassed *gpd*, and *eno* had the tendency to do so at higher glucose concentrations. Therefore, the promoters of *pdc* and *eno* were selected for the construction of constitutive expression cassettes, and the *gpd* promoter was selected as the reference. The promoter and the terminator regions of these genes were acquired from the genome sequence of *T. reesei* (http://genome.jgi-psf.org/Trire2/Trire2.home.html). Ppdc, the promoter of *pdc*, is located from 106110 bp to 107643 bp in scaffold 8; Tpdc, the terminator of *pdc*, is located from 103156 bp to 104185 bp in scaffold 8. Peno, the promoter of *eno*, is located from 102421 bp to 103910 bp in scaffold 4; Teno, the terminator of *eno*, is located from 99879 bp to 100865 bp in scaffold 4. Pgpd, the promoter of *gpd*, is located from 1561323 bp to 1562759 bp in scaffold 1; and Tgpd, the terminator of *gpd*, is located from 1564087 bp to 1564995 bp in scaffold 1.

### Application of strong promoters in homologous xyn2 expression

**Figure 3:**
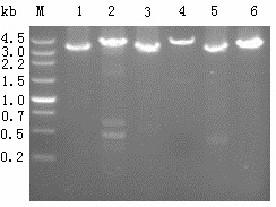
**Agarose gel electrophoresis analysis of PCR amplified XYNII expression cassettes from the recombinant strains.***Lanes*: *M* the DNA molecular weight marker, *Lanes 1, 3, 5* the PCR amplified Peno-xyn2-Teno expression cassette (3282 bp), Ppdc-xyn2-Tpdc expression cassette (3369 bp), and Pgpd-xyn2-Tgpd expression cassette (3151 bp); *Lanes 2, 4, 6* the native expression cassettes for *eno* (4032 bp), *pdc* (4488 bp), and *gpd* (3673 bp).

To evaluate the transcriptional activities of the promoters screened by RT-qPCR, x*yn2* was selected as a reporter, since it is convenient and standardized to perform xylanase activity assays. This gene encodes XYNII, a small xylanase of *T. reesei* that has been expressed in various hosts [20-22]. The *xyn2* expression cassettes, Ppdc-xyn2-Tpdc, Peno-xyn2-Teno, and Pgpd-xyn2-Tgpd, were respectively mixed with pAN7-1, and the mixtures were used to transform *T. reesei* QM9414. About 25% of the transformants that were resistant to hygromycin B contained the co-transformed expression cassette. The transformants that exhibited the highest xylanase productivity for each expression cassette were designated as *T. reesei* pxyn2, *T. reesei* exyn2, and *T. reesei* gxyn2, respectively. The expression cassettes in the transformants were PCR amplified, with the genomic DNA as the template (Figure 3), and the PCR amplified products were sequenced and proven to be correctly constructed. Southern blot analysis showed that all of these transformants had a single-copy insert of the expression cassette. The expression cassettes might be inserted into the genome of *T. reesei* randomly, for the endogenous *pdceno* and *gpd* cassettes remained intact in the transformants (Figure 3).

**Figure 4:**
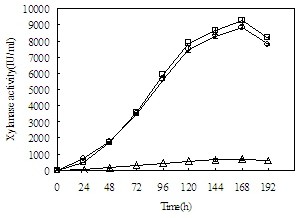
**Time course of recombinant xylanase II production by the recombinant strains.** □: *T. reesei* pxyn2, ○: *T. reesei* exyn2, ▵: *T. reesei* gxyn2. Error bars represent standard deviations.

Then, *T. reesei* pxyn2, *T. reesei* exyn2, and *T. reesei* gxyn2, which respectively expressed *xyn2* under the control of promoters Ppdc, Peno, Pgpd, were cultivated for recombinant XYNII production. The parent strain, *T. reesei* QM9414, was selected as the control. The activity of xylanase in the cultivation supernatant of the three transformants all reached the peak at 168 h of incubation (Figure 4). *T. reesei* pxyn2 exhibited the highest productivity of recombinant XYNII, with 9266 IU/ml of xylanase activity in the culture supernatant, while *T. reesei* exyn2 produced 8866 IU/ml of xylanase activity, which is slightly lower than that of *T. reesei* pxyn2. *T. reesei* gxyn2, in which the transcription of recombinant *xyn2* was controlled by Pgpd, produced 686 IU/ml of xylanase activity, approximately 7% of that of *T. reesei* pxyn2. Hardly any production of XYNII could be detected in *T. reesei* QM9414.

**Figure 5:**
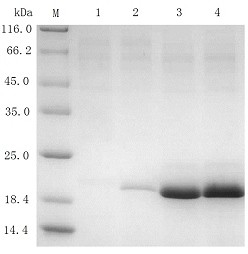
**SDS-PAGE analysis of expressed xylanase II in the culture supernatant of the recombinant strains.***Lanes: M* low molecular marker, *1* the parent strain *T. reesei* QM9414, *2* strain *T. reesei* gxyn2, *3* strain *T. reesei* exyn2, *4* strain *T. reesei* pxyn2. Equal amounts of protein (3 μl supernatant) are applied in each lane.

The proteins in the culture supernatant of the recombinant strains were analyzed by SDS-PAGE (Figure 5). Recombinant XYNII secreted by *T. reesei* pxyn2 and *T. reesei* exyn2 was the most abundant protein in the supernatant, accounting for approximately 83% and 82% of the total protein as estimated by the densitometry method. The amount of total protein in the supernatant of *T. reesei* pxyn2 and *T. reesei* exyn2 was 1.94 g/L and 1.85 g/L, respectively, as determined by the Bradford method. Therefore, the recombinant XYNII secreted by *T. reesei* pxyn2 and *T. reesei* exyn2 strains was 1.6 g/L and 1.5 g/L, respectively. As for the recombinant strain *T. reesei* gxyn2, the secreted recombinant XYNII was still the most prominent protein, but far less abundant than that of the other two recombinant strains. In agreement with the results of the xylanase activity assay, there was no visible protein band that corresponded to XYNII in the SDS-PAGE gel of the parent strain *T. reesei* QM9414. The results showed that the strong constitutive promoters screened by RT-qPCR have great potential in conducting recombinant gene expression in *T. reesei*.

## Discussion

The strong *cbh1* promoter has been used frequently for heterologous or homologous protein expression in *T. reesei*. However, this promoter needs induction and is at least partly regulated by catabolite repression. Several constitutive promoters, such as the *tef1* and *pyk* promoters, have been used to drive recombinant protein production in *T. reesei*, but their transcriptional activities are fairly low when compared with the *cbh1* promoter [15,17]. In general, there is still a lack of constitutive promoters for *T. reesei*, either for heterologous protein production or for genetic manipulation. In yeast, there are a number of research works dealing with promoter finding and optimization, and many effective promoters have been characterized, such as the GAP promoter in *Pichia pastoris* and the TEF1 promoter in *Saccharomyces cerevisiae*[23-25]. This report has analyzed the transcriptional efficiency of the promoters of 13 key genes that participate in glucose metabolism through RT-qPCR. The results indicate that the *pdc* promoter (Ppdc), the *eno* promoter (Peno), and the *gpd* promoter (Pgpd) are more active in glucose containing medium. Glucose concentration influences the transcriptional efficiency of the promoters in different ways, i.e., the transcriptional efficiency of Ppdc and Peno dramatically increases at high glucose concentrations, while that of Pgpd only increases slightly.

To verify the actual ability of these promoters in triggering recombinant protein production, expression cassettes of *xyn2* with the promoters were constructed and transformed into *T. reesei* QM9414, respectively. The recombinant strains, *T. reesei* pxyn2, *T. reesei* exyn2 and *T. reesei* gxyn2 for the *pdc, eno* and *gpd* promoters, respectively, were cultivated in a modified Mandels medium with a glucose concentration of 7%. The *T. reesei* pxyn2 strain presented the highest ability to produce recombinant protein, while the *T. reesei* gxyn2 strain had a lower ability. This result is consistent with the data of the expression levels of the corresponding genes initiated by these promoters as analyzed through RT-qPCR. Although the mRNA level of *pdc* is lower than *gpd* at low glucose concentrations, it increases faster with glucose concentration, and at 85 mM of glucose concentration, the mRNA level of *pdc* is 2.1 times higher than the *gpd*. The mRNA level of *eno* also shows the trend to increase faster with glucose concentration, and it is reasonable that the productivity of recombinant xylanase of *T. reesei* exyn2 is 8866 IU/ml, which is also much higher than *T. reesei* gxyn2. As indicated in Figure 2, the *gpd* promoter is more efficient than the *eno* promoter when the glucose concentration is 85 mM. However, glucose concentration in the medium for recombinant xylanase production is 7%, which is approximately 388 mM and much higher than 85 mM. Therefore, there is enough room for the efficiency of the *eno* promoter to increase and surpass that of the *gpd*.

Aside from cellulase, *T. reesei* is also regarded as an important producer of xylanase. Induced by arabinose-rich plant hydrolysates and lactose in fed-batch cultures, the mutant strain *T. reesei* Rut C-30 produces up to 1350 IU/ml of xylanase [26]. However, under induced conditions, *T. reesei* produces a large amount of cellulase aside from xylanase, and cellulase is problematic for the application of xylanase in some industrial processes, such as biobleaching [27]. To resolve this problem, many researchers have utilized a heterologous expression system to produce xylanase from recombinant fungi or bacteria. For example, a xylanase gene (*xyn6*) originated from the thermophilic fungus *Humicola grisea* has been cloned and expressed in *T. reesei* by Nevalainen *et al.*[28,29], and a productivity of 0.5-1 g/L recombinant xylanase is achieved. In the present study, we screened strong constitutive promoters of *T. reesei* through RT-qPCR, and developed a novel approach to produce cellulase-free xylanase by using the promoters of the *pdceno* and *gpd* genes. The *T. reesei* pxyn2 and *T. reesei* exyn2 recombinant strains, in which the *pdc* and the *eno* promoters were respectively used to conduct the *xyn2* gene expression, produced 9266 IU/ml and 8866 IU/ml of activity in a modified Mandels medium. Moreover, under constitutive production with high glucose concentrations, the recombinant strains produce little other proteins as revealed by the SDS-PAGE image (Figure 5), and only a trace of cellulase activity is detected, with 0.3 IU/ml of CMCase activity and 0.03 FPIU/ml of filter paper activity. To date, the highest native production of obtained in *T. reesei* is 1800 IU/ml with a fed-batch cultivation process [26], and the highest heterologous production of xylanase obtained is 3676 IU/ml with a fed-batch cultivation of *Pichia pastoris* expressing *A. niger xylB*[30]. We used strong constitutive promoters in the xylanase production in *T. reesei*, and obtained recombinant productivity of 9266 IU/ml for the *pdc* promoter and 8866 IU/ml for the *eno* promoter in batch cultivation, which are much higher than the best native and heterologous xylanase production levels to date. Thus, this study has reported the most efficient process for xylanase production among all native and heterologous production processes.

## Conclusions

The expression efficiencies of the genes related with glucose metabolism have been analyzed through RT-qPCR. The results reveal that the *pdc* promoter and the *eno* promoter are highly active, especially in medium with high concentration of glucose. These promoters have great potential in driving recombinant gene expression in *T. reesei*. Two recombinant strains, *T. reesei* pxyn2 and *T. reesei* exyn2, that respectively contained the *xyn2* expression cassettes constructed with the promoters of *pdc* and *eno*, exhibit high productivity of recombinant XYNII in medium with high concentration of glucose. In addition, recombinant XYNII is the dominant protein in the culture supernatant, and the cellulase activity produced is negligible. The approach of producing recombinant proteins in *T. reesei* with the promoters high functional on glucose could be widely applied in industrial enzyme production.

## Materials and methods

### Strains, plasmids, and cultivation conditions

*Escherichia coli* (*E. coli)* Top10F’ (Invitrogen, USA) was used for plasmid construction and maintenance. *T. reesei* QM9414 (ATCC 26921) was used as a parental strain throughout the study. The *E. coli* strain was cultivated in LB medium, in which ampicillin (100 μg/ml, Invitrogen) was supplemented when necessary. The *T. reesei* strain was maintained on potato dextrose agar (PDA), and for liquid cultivation, it was grown in Mandels medium that contained an appropriate concentration of glucose [31]. The recombinant *T. reesei* strains were selected on PDA agar supplemented with hygromycin B (100 μg/ml), and for recombinant xylanase production, the strains were cultivated in modified Mandels medium supplemented with 7% glucose, 5% soybean powder, and 1% peptone. The *E. coli* and *T. reesei* strains were routinely cultured at 37°C and 28°C, respectively. Plasmid pUC19 was used for the construction of *xyn2* expression cassettes. Plasmid pAN7-1 which contained the hygromycin B resistant cassette was used as an assisting plasmid for the transformation of *T. reesei*[32].

### RNA extraction and cDNA synthesis

About 10^7^*T. reesei* spores collected from a PDA plate grown for 5 days were inoculated into a 2-liter flask that contained 400 ml of Mandels medium with glucose at a final concentration of 1.8% (100 mM). They were then grown at 28°C and 250 r/min. Samples were taken at 24, 44 and 84 hours. Mycelia were harvested by centrifuge, frozen in liquid nitrogen and stored at −80°C. The glucose concentration in the samples was measured by using the 3, 5-dinitrosalicylic acid (DNS) method [33]. The total RNA of the samples was extracted by using a Universal Plant Total RNA Extraction Kit (BioTeke Corporation, China). To remove the genomic DNA, the RNA preparations were treated with DNase I (Fermentas, Canada). The quantity and quality of the extracted RNA were assessed on a GeneQuant 1300 spectrophotometer (Biochrom Ltd., England) and by agrose gel electrophoresis. The synthesis of the complementary DNA (cDNA) from 1.0 μg of the total RNA per reaction (20 μl) was carried out by using a PrimeScript reagent kit (TaKaRa).

### Quantitative real-time PCR

Quantitative real-time PCRs (RT-qPCRs) were performed in an ABI Prim 7300 System (Applied Biosystems, USA). Each reaction mixture contained 2 μl of template (1:60 dilution of the reverse transcription (RT) reaction product), 10 μl of SYBR Premix Ex Taq 2× (TaKaRa), 300 nmol/L of forward and reverse primers (Table 1), and nuclease-free water with a final volume of 20 μl. The PCR protocol consisted of 30 s of initial denaturation at 95°C, followed by 40 cycles of 5 s at 95°C and 31 s at 60°C. A melting curve was performed after each run to check the PCR product specificity. All PCRs were carried out in triplicate on a plate. Data obtained by using the ABI Prim 7300 Sequence Detection System were analyzed as described in the Applied Biosystems User Bulletin #2. The expression levels of the genes in the glucose metabolism were normalized with an endogenous control, *sar1*, as previously described by Steiger *et al.*[34]. The means ± standard deviations of replicates are shown in the figures.

**Table 1:** Sequences of primers used in cDNA synthesis

**Name**	**Sequence (5′–3′)**	**Amplification target**
sar-1	tggatcgtcaactggttctacga	*sar1* cDNA
sar-2	gcatgtgtagcaacgtggtcttt	
gpd-F	gtgctgcccagaacatcatcc	*gpd* cDNA
gpd-R	tggcggtagggacacgaatg	
pdc-F	ttcaacactgcgggcttct	*pdc* cDNA
pdc-R	cagaacacccctcatcgctac	
kdh-F	aggaaaccgaggagacatatacc	*kdh* cDNA
kdh-R	gctcaaggacgagttggaaatg	
pda-F	aacctccctgctctgtttggc	*pda* cDNA
pda-R	tcctcgcttgtagtagtcggtca	
eno-F	agcttgccgccatgtactctg	*eno* cDNA
eno-R	aatctggatgtcctgggtcttg	
ald1-F	agaagacggagcagtagttgagg	*ald1* cDNA
ald1-R	tgagaagattgtgggttggatg	
glk-F	aagccaacgcgggattaaag	*glk* cDNA
glk-R	gtgccgcccaggtctacag	
pyk-F	cctcagcatcaaggacgaccag	*pyk* cDNA
pyk-R	acatcggtgttgggcaggttg	
cit-F	ggacctcctccgtctctaccttg	*cit* cDNA
cit-R	ggtcactcagggcactaccaaca	
adh-F	cttccagcaggcaaccgagt	*adh* cDNA
adh-R	tggtgatcatgcggatgacg	
fba-F	cctgtgcggtatccgtgact	*fba* cDNA
fba-R	ggacatggtcttctcaccctcg	
tpi-F	aacgagaagaactgcggtgaac	*tpi* cDNA
tpi-R	gacgatgtcaatgaaggcagg	
ald2-F	gagggcaagacgctggaca	*ald2* cDNA
ald2-R	gcatgagcagggggaagttc	

### Construction of Ppdc-xyn-Tpdc expression cassette

The promoter of the *pdc* gene (Ppdc, 1,534 bp upstream fragment starting from the start coden of *pdc*), the *xyn2* gene, and the terminator of the *pdc* gene (Tpdc, 1,030 bp downstream fragment starting from the stop codon of *pdc*) were PCR amplified by using the genomic DNA of *T. reesei* QM9414 as the template. The primers Ppdc-F and Ppdc-R were used for amplification of Ppdc, and a *Hin*dIII restriction site was added to the 5’-end of the amplified product. The primers xyn-p-F and xyn-p-R were used for amplification of *xyn2* (GenBank accession number: U24191.1), and a *Pst*I site was added to the 3’-end. The signal peptide coding sequence was included in the amplified *xyn2*. The primers Tpdc-F and Tpdc-R were used for Tpdc amplification, and *Pst*I and *Xba*I sites were added to the 5’ and 3’ ends, respectively. The amplified Ppdc and *xyn2* fragments were fused by overlapping extension PCR through the use of primers Ppdc-F and xyn-p-R, and the resulting fusion fragment was inserted into pUC19 by using the restriction sites *Hin*dIII and *Pst*I, which generated recombinant plasmid pUC19-Ppdc-xyn2. Finally, the Tpdc fragment was inserted into plasmid pUC19-Ppdc-xyn2 by using *Pst*I and *Xba*I sites, which generated plasmid pUC19-Ppdc-xyn2-Tpdc. This plasmid was used for the transformation of *T. reesei* QM9414 and the recombinant expression of XYNII. The primers are listed in Table 2. The restriction enzymes, DNA polymerase and T4 DNA ligase were purchased from TaKaRa.

**Table 2:** Sequences of primers used for construction of expression cassettes

**Name**	**Sequence (5′–3′)**	**Amplification target**
Ppdc-F	cccaagcttaggacttccagggctacttg (*Hind*III)	*pdc* promoter
Ppdc-R	gattgtgctgtagctgcgct	
xyn-p-F	atggtctccttcacctccctc	*xyn* coding sequences
xyn-p-R	gcaactgcagcacatcacaaaagaagagcccc (*Pst*I)	
Tpdc-F	gcaactgcagcccggcatgaagtctgacc (*Pst*I)	*pdc* terminator
Tpdc-R	gctctagatggacgcctcgatgtcttcc (*Xba*I)	
Peno-F	gcaactgcagtgattccgtcctggattgc (*BamH*I)	*eno* promoter
Peno-R	tttgaagctatttcaggtggctgg	
xyn-e-F	atggtctccttcacctccctc	*xyn* coding sequences
xyn-e-R	ggggtacccacatcacaaaagaagagcccc (*Kpn*I)	
Teno-F	ggggtaccatggccacgagagacaactacc (*Kpn*I)	*eno* terminator
Teno-R	ggaattctggcgtcgttgatgtttcg (*EcoR*I)	
Pgpd-F	gctaagcttgacgcagaagaaggaaatcgcc (*Hind*III)	*gpd* promoter
Pgpd-R	tttgtatctgcgaattgagcttgcg	
xyn-g-F	atggtctccttcacctccctc	*xyn* coding sequences
xyn-g-R	gcaactgcagcacatcacaaaagaagagcccc (*Pst*I)	
Tgpd-F	gcaactgcaggtgctgtgttcctcagaatggg (*Pst*I)	*gpd* terminator
Tgpd-R	gctctagattacggatctgatcactcggg (*Xba*I)	

*Restriction sites are underlined by single lines, and overlapping regions for overlap extension PCR are indicated by double lines. *F*: forward primer, *R*: reverse primer.

### Construction of Peno-xyn-Teno expression cassette

This procedure is similar to the construction of the Ppdc-xyn-Tpdc expression cassette. The *eno* promoter (Peno, 1,490 upstream fragment starting from the start codon of the *eno* gene), *xyn2* gene, and *eno* terminator (Teno, 987 bp downstream fragment starting from the stop codon of the *eno* gene) were PCR amplified from *T. reesei* QM9414 genomic DNA with the primers listed in Table 2. Restriction sites were added to the fragments as needed. The amplified Peno and *xyn2* fragments were fused by overlapping extension PCR through the use of primers Peno-F and xyn-e-R, and the resulting fusion fragment was inserted into pUC19. Finally, Teno was inserted into the plasmid, which generated the expression plasmid pUC19-Peno-xyn2-Teno.

### Construction of Pgpd-xyn-Tgpd expression cassette

For construction of the Pgpd-xyn-Tgpd expression cassette, the *gpd* promoter (Pgpd, 1,437 bp upstream fragment starting from the start codon of the *gpd* gene), *xyn2* gene, and *gpd* terminator (Tgpd, 805 bp downstream fragment starting from the stop codon of the *gpd* gene) were PCR amplified from *T. reesei* QM9414 genomic DNA with the primers listed in Table 2. Restriction sites were added to the fragments as needed. The amplified Pgpd and *xyn2* fragments were fused by overlapping extension PCR through the use of primers Pgpd-F and xyn-g-R, and the resulting fusion fragment was inserted into pUC19. Finally, Tgpd was inserted into the plasmid, which generated the expression plasmid pUC19-Pgpd-xyn2-Tgpd.

### Protoplast transformation of T. Reesei

Protoplast transformation of *T. reesei* was performed by using the polyethylene glycol method as described in Punt *et al.*[32]. Lysing enzymes from *Trichoderma harzianum* (Sigma-Aldrich) were used in the *T. reesei* protoplast preparation. For the transformation, the expression cassettes were released from the plasmids through digestion with appropriate restriction enzyme pairs, then purified, and mixed with equal amounts of plasmid pAN7-1. The mixture was then used for co-transformation of *T. reesei* protoplasts. Candidate transformants were streaked twice on PDA plates that contained 100 μg/ml of hygromycin B, and then transferred to PDA plates to form conidia. For each expression cassette, twenty single colonies were selected for cultivation in Mandels medium supplemented with 4% glucose, and the activity of xylanase in the supernatant was analyzed. For each expression cassette, the single colony that exhibited the highest productivity was selected for further study.

### Southern blot hybridization

The chromosomal DNA was extracted and purified by the phenol/chloroform method. The DNA was digested with *Sac*I and *Bam*HI, fractionated on 0.7% (w/v) agarose gels and then transferred to nylon membranes (Roche). High-stringency probing was carried out at 50 °C overnight using digoxigenin (DIG)-labeled DNA probes, which were produced by amplifying a 494 bp fragment of the *T.reesei xyn2* gene with the primers Probe-F (aatccgtggctgtggagaag) and Probe-R (tgcgtgcggtaaatgtcgta) and labeled with digoxigenin DNA labeling mix (Roche). Chromogenic signal detection was done with the detection system from Roche Molecular Biochemicals. NBT/BCIP was used as the Chromogenic substrate.

### Enzyme assays and protein analysis

For recombinant xylanase production, about 10^5^ spores of the recombinant *T. reesei* strains were inoculated into 30 ml of Mandels medium, and maintained at 28°C and 250 r/min for 48 h. Then, 1.5 ml of the above culture was transferred into 30 ml of a modified Mandels medium, and maintained at 28°C and 250 r/min for about 192 h. In the modified minimal medium, the glucose concentration was raised from 2% to 7%, concentration of peptone from 0.5% to 1%, and 5% soybean powder was added. Xylanase activity was assayed as described in Bailey et al. with birchwood xylan (Sigma-Aldrich) as the substrate [35]. The culture supernatant was assayed for total cellulase activity as previously described by using two different substrates: 2% (w/v) carboxy methyl cellulose (CMC) and filter paper (Whatman No.1) [36]. One unit of enzyme activity (IU) was defined as the amount of enzyme that released 1 μmol reducing sugar per minute at 50°C.

Total protein concentration in the culture supernatant was determined by using a Bradford reagent kit (Sangon Biotech, China). Sodium dodecyl sulfate- polyacrylamide gel electrophoresis (SDS-PAGE) was performed in 12.5% polyacrylamide gel slabs as described in Laemmli [37]. Three μl of each sample was mixed with 10 μl sample-loading buffer, boiled for 5 min, and loaded into the sample well. Proteins were stained with Coomassie Brilliant Blue R-250 (Sangon Biotech, China). The amount of protein in the SDS-PAGE bands was estimated by densitometry through the use of a Furi FR-200A ultraviolet analyzer (Furi Tech, China).

## Competing interests

The authors declare that they have no competing interests

## Author’s contributions

GL and JW designed the experiments and wrote the manuscript. JXL performed most of the experiments. SWW participated in RT-qPCR experiments and fugal transformation. MX participated in enzyme and protein concentration assay. SWY participated in construction of expression cassettes. All authors read and approved the final manuscript.
